# Revaluating the Dimensionality of Academic Engagement: A Bifactor Analysis of the UWES in Higher Education

**DOI:** 10.3390/bs16071045

**Published:** 2026-06-23

**Authors:** Alejandro Vega-Muñoz, Beatriz Sora, Joan Boada-Grau, David Chavez-Herting, Natalia Salas-Guzmán

**Affiliations:** 1 Facultad de Ciencias Empresariales, Universidad Arturo Prat, Santiago 8340232, Chile; alejavega@unap.cl; 2 Facultad de Medicina y Ciencias de la Salud, Universidad Central de Chile, Santiago 8330507, Chile; 3 Faculty of Education Sciences and Psychology, University Rovira i Virgili, 43007 Tarragona, Spain; beatriz.sora@urv.cat (B.S.); joan.boada@urv.cat (J.B.-G.); 4 Facultad de Educación y Ciencias Sociales, Universidad Finis Terrae, Santiago 7501015, Chile

**Keywords:** Utrecht Work Engagement Scale, student well-being monitoring, university students, Chile, bifactor analysis

## Abstract

The factor structure of the Utrecht Work Engagement Scale (UWES) has been debated, with studies alternately supporting unidimensional and three-factor solutions. This inconsistency may reflect a methodological limitation: conventional confirmatory factor analysis (CFA) cannot always separate general from dimension-specific variance, producing similar fit indices across competing models when a dominant general factor is present. We examined the dimensionality of the UWES-17 and UWES-9 in a sample of 755 Chilean university students, comparing unidimensional, three-factor, second-order, and bifactor models using weighted least squares mean and variance adjusted (WLSMV) estimation appropriate for ordinal data. Bifactor indices, explained common variance (ECV), percent of uncontaminated correlations (PUC), and hierarchical omega (ω_h_), were computed to evaluate essential unidimensionality. Results indicated that a general engagement factor explained approximately 85% of common item variance in both versions (ECV ≈ 0.85; ω_h_ > 0.90), while specific factors for vigor, dedication, and absorption retained negligible reliable variance, particularly absorption (ω_h_ ≈ 0.00). Measurement invariance by sex was supported for the UWES-9 at the metric level, whereas classical UWES-17 solutions showed instability, including factor collapse and non-convergence of the second-order model. Taken together, findings suggest that the apparent multidimensionality of the UWES may be, at least partly, an artifact of conventional CFA modeling rather than a substantive property of the construct in this student sample. For applied monitoring of student well-being, the UWES-9 total score appears to be the most pragmatic and psychometrically defensible approach for assessing general academic engagement in this Chilean university sample, while institutional well-being monitoring would ideally be further supported by criterion-related, predictive, and sensitivity-to-change evidence.

## 1. Introduction

Work engagement has emerged over recent decades as a central construct in organizational and educational psychology, associated with multiple indicators of well-being, performance, and psychological adjustment. It has traditionally been defined as a positive, persistent, work-related state of mind characterized by three core components: vigor, dedication, and absorption (Schaufeli et al., 2002). This conceptualization served as the foundation for the development of the Utrecht Work Engagement Scale (UWES), one of the most widely used instruments for assessing engagement across diverse contexts (Schaufeli et al., 2002).

Beyond its motivational nature, work engagement has consistently been linked to indicators of psychological well-being, such as positive affect, life satisfaction, and lower levels of distress, positioning it as a key component of positive mental health rather than merely a performance-related construct (Schaufeli et al., 2002, 2006). In educational settings, student engagement has been associated with better academic adjustment, higher satisfaction with studies, and reduced risk of dropout, highlighting its role as a protective factor for student mental health and overall academic functioning, particularly in contexts of elevated academic stress and psychological demands.

In the university setting, engagement has established itself as a positive indicator of student well-being and academic adjustment, as it reflects a motivational emotional state characterized by vigor, dedication, and absorption. Evidence shows that students who are more engaged and energized tend to exhibit higher levels of well-being, lower emotional exhaustion, and more adaptive academic functioning (Carmona-Halty et al., 2019; Tayama et al., 2019). Additionally, research indicates that engagement contributes to better academic performance through motivational pathways, reinforcing its relevance for understanding students’ psychological functioning (Salanova et al., 2010; Usán Supervía et al., 2020). From this perspective, the UWES not only assesses a motivational state but also serves as a relevant indicator of positive psychological well-being, supporting its use as a positive mental-health indicator in institutional efforts to monitor student well-being, prevent burning out, and promoting healthier and more sustainable academic trajectories. However, in the present study, we focus primarily on the structural validity of UWES scores, and institutional applications for well-being monitoring are discussed as potential uses that would benefit from additional criterion-related evidence.

The UWES was originally developed in its 17-item version (UWES-17), organized around three dimensions reflecting the theoretical components of the construct. A nine-item abbreviated version (UWES-9) was subsequently proposed for reasons of parsimony and psychometric stability, demonstrating adequate properties across diverse studies and cultural contexts (Schaufeli et al., 2006; Kulikowski, 2017). In this sense, the UWES can be understood not only as a measure of motivational engagement, but also as a positive indicator of mental health, given its conceptual opposition to burnout and its empirical association with indicators of psychological well-being. The instrument has also been adapted to educational settings through student-specific versions, most notably the Utrecht Work Engagement Scale for Students (UWES-S), which preserves the vigor–dedication–absorption structure while applying items to academic activities (Schaufeli et al., 2002; Valdez-Bonilla et al., 2011). In the present study, we used the UWES-S rather than the occupational UWES, extending its use in research on academic engagement (Jang & An, 2022; Rodríguez-González et al., 2023).

From a higher education perspective, valid and reliable measures of engagement are crucial for monitoring student well-being and informing institutional strategies. Universities increasingly face challenges related to stress, burnout, and academic disengagement, which are associated with mental health problems and increased risk of academic failure and dropout. In this context, instruments such as the UWES-Student version offer a positive, resource-oriented indicator that can complement risk-focused screening tools and inform the development of evidence-based policies for student support, mental health promotion, retention, and the early detection of psychological risk.

Despite its widespread adoption, the factor structure of the UWES remains an unresolved issue in literature. In a review of 21 studies, Kulikowski (2017) found that the original three-factor structure was superior in six of them, the unidimensional model performed best in another six, and both structures showed equivalent fit in eight studies. This near-even split illustrates the persistent lack of consensus on the instrument’s dimensionality; accumulated evidence offers no clear resolution (Kulikowski, 2017). At the same time, bibliometric and mapping reviews of academic engagement research highlight the rapid growth and conceptual diversity of engagement studies in educational settings, which helps explain the proliferation of differing measurement approaches and the need for clearer conceptual boundaries when engagement is used as an indicator of student well-being and mental-health status. (Loyola-Carrillo et al., 2025; Truta et al., 2018).

One likely explanation for this inconsistency is the high intercorrelations among UWES dimensions. Prior research has consistently reported substantial correlations between vigor, dedication, and absorption across samples from different countries and cultural contexts, raising questions about their empirical discriminability and suggesting the presence of a dominant general factor (Kulikowski, 2017; Merino-Soto et al., 2022). Some authors have consequently opted for a global engagement score, while others have maintained the use of subscales based on specific analytic objectives (Schaufeli et al., 2006). However, this high covariance is not always adequately captured by traditional CFA models, which can lead to erroneous conclusions about the structure of the instrument—a limitation also highlighted in the broader research on engagement, where it has been shown that CFA tends to inflate correlations between factors and obscure multidimensionality (Lee, 2021). Recent empirical studies in student populations also document similar measurement challenges and emphasize the need to consider ordinal estimation and bifactor approaches (Chi et al., 2023; Ng et al., 2022).

Additionally, methodological choices in prior studies may also contribute to this variability. In particular, the use of maximum likelihood estimators with ordinal data, and the introduction of residual correlations guided by modification indices, can improve fit indices without necessarily reflecting a better theoretical representation of the construct. Indeed, some studies have reported excellent fit for multiple competing factor models, which may indicate the limited discriminative capacity of CFA under certain data conditions, especially when engagement is used for well-being monitoring rather than purely motivational assessment (Santalla-Banderali & Alvarado, 2022). Methodological reviews and psychometric investigations recommend robust ordinal estimators (e.g., WLSMV) and the computation of bifactor indices (ECV, ω_h_, PUC) to evaluate essential unidimensionality (Rodriguez et al., 2016; Merino-Soto et al., 2022).

Within the Latin American context, empirical evidence on the UWES’s factor structure remains limited. In Chile, Carmona-Halty et al. (2019) evaluated the abbreviated version in university students, finding support for the three-factor structure and evidence of sex-based measurement invariance. In Peru, Merino-Soto et al. (2022) applied bifactor modeling to the UWES-15 and UWES-9 in a worker sample, concluding that a general factor predominantly explains common item variance and that the dedication dimension was entirely absorbed by this general factor, losing statistical autonomy. Most of these studies, however, have examined a restricted set of models or specific samples, without systematically comparing different structural specifications or examining their implications for student well-being assessment in the Chilean context using both versions of the instrument (Carmona-Halty et al., 2019; Merino-Soto et al., 2022). Recent Latin American psychometric work supports the utility of bifactor analyses to clarify whether subscales retain meaningful unique variance (Merino-Soto et al., 2022). Thus, the contribution of the present study is not limited to examining a Chilean university sample. Its added value lies in the simultaneous comparison of the UWES-17 and UWES-9, the use of WLSMV estimation for ordinal indicators, the deliberate avoidance of post hoc residual modifications, and the reporting of bifactor indices to determine whether total-score or subscale-score interpretation is psychometrically warranted.

While the UWES demonstrates validity evidence across diverse contexts, important questions persist regarding its factor structure. Prior studies have shown that high inter-factor covariation can compromise the discriminability of vigor, dedication, and absorption, and that traditional CFA models may not adequately capture this complexity, leading to erroneous conclusions about the instrument’s structure, a limitation also highlighted in broader engagement research showing that CFA tends to inflate factor correlations and obscure multidimensionality (Lee, 2021).

In response to these limitations, the bifactor model has gained prominence as a tool for addressing this question. It decomposes item variance into a general factor, capturing variance shared across all items, and specific factors representing each dimension’s unique variance. This approach is particularly suited to instruments with high inter-factor covariation, as it simultaneously estimates whether a dominant general construct exists and to what extent dimensions retain interpretable specific variance (Rodriguez et al., 2016). Recent studies applying this model to the UWES have consistently found that the general factor explains the majority of common item variance, both in Latin American worker samples (Merino-Soto et al., 2022) and in samples from other regions (Sanhokwe, 2022; L. Hu et al., 2023), casting doubt on the utility of computing and interpreting separate subscale scores, particularly when institutions rely on engagement as a mental-health indicator and academic support policies. Complementary student-focused research also links engagement scores to academic outcomes (performance, dropout intentions, well-being), reinforcing the practical importance of clarifying scoring decisions (Truta et al., 2018; Villacís et al., 2021; Gavín-Chocano et al., 2024).

Clarifying the dimensionality of the UWES in university students is therefore not only a psychometric issue, but also a matter of practical relevance for student mental health and institutional decision-making. If engagement can be validly summarized in a robust general factor, universities may rely on a single, interpretable indicator to monitor student well-being, identify at-risk groups, and evaluate the impact of well-being and academic support policies. Evidence from other educational contexts also shows that overall engagement is the strongest predictor of academic outcomes, reinforcing the practical value of clarifying its role (Shernof et al., 2017; Sjogren et al., 2022). Conversely, if specific dimensions retain meaningful unique variance, more differentiated profiles of student functioning could inform targeted interventions in areas such as energy management, sense of purpose, immersive study experiences, and psychological support strategies.

This study systematically examines the factor structure of the UWES in a university student sample by comparing four model specifications across both the 17-item and 9-item versions: unidimensional, three-factor, second-order, and bifactor. The bifactor model is included to directly assess whether a dominant general engagement factor accounts for most common item variance and whether specific dimensions retain sufficient independent variance to warrant separate interpretation. Unlike prior studies focused on workers, the analysis uses a Chilean student sample, robust ordinal estimation, and no post hoc model modifications. This study adds to prior work by simultaneously comparing the UWES-17 and UWES-9 in Chilean university students, using WLSMV estimation for ordinal data, deliberately avoiding post hoc model modifications, and reporting bifactor indices (ECV, PUC, ω_h_) to inform scoring decisions. These features distinguish the present contribution within the Latin American and higher-education engagement literature.

The study follows methodological recommendations for reporting bifactorial indices (e.g., ECV, PUC, ω_h_) and evaluating the practical implications of different scoring strategies in educational research and practice (Rodriguez et al., 2016). Accordingly, the central applied question is whether the UWES should be interpreted primarily through a total engagement score or whether vigor, dedication, and absorption retain sufficient reliable specific variance to justify separate subscale reporting. This distinction is important because a three-factor CFA model may show acceptable or even superior fit while still providing limited evidence that the subscales contain meaningful reliable variance beyond a dominant general factor.

## 2. Materials and Methods

### 2.1. Design and Participants

This study employed a cross-sectional design to examine the factor structure of the Utrecht Work Engagement Scale (UWES) through confirmatory factor analysis. The sample comprised 755 university students from the Metropolitan Region of Chile, selected through non-probabilistic convenience sampling. Of the total participants, 375 identified as male (49.7%) and 380 as female (50.3%), with a mean age of 21.63 years (SD = 5.59; range: 18–70). While the mean and standard deviation reflect a predominantly young sample, 40 participants older than 30 years (5.3%) were observed, with ages reaching up to 70 years. No exclusions were made based on age, as this variable was not used as a segmentation criterion or as a predictor in the present analyses. The study aimed to determine whether the UWES behaves as a multidimensional or essentially unidimensional construct in university students.

### 2.2. Instrument

Work engagement was assessed using the Utrecht Work Engagement Scale for Students (UWES-S), the student-adapted version in Spanish of the UWES developed by Schaufeli and Bakker (Valdez-Bonilla et al., 2011). The UWES-S preserves the original three dimensions (vigor, dedication, absorption) but applies them to academic activities. In the present study, both the 17-item (UWES-17S) and 9-item (UWES-9S) student versions were administered. In this study, the UWES-9 was derived from the UWES-17 item pool by selecting the three items per dimension that correspond to the abbreviated version, following Schaufeli et al. (2006). UWES items were rated on a Likert-type frequency scale with 7 response categories, ranging from 0 (“never”) to 6 (“always”), which reinforces the treatment of observed variables as ordinal in the subsequent analyses.

This instrument was developed within the positive psychology framework as a conceptual alternative to burnout research, with the goal of assessing positive occupational well-being states independently (Schaufeli & Bakker, 2001). In the present study, “academic engagement” is conceptualized as the student-specific manifestation of work engagement, preserving the vigor, dedication, and absorption dimensions while applying them to academic activities.

Work engagement is defined as a positive, persistent, work-related state of mind characterized by three dimensions: vigor, dedication, and absorption (Schaufeli et al., 2002). Vigor refers to high levels of energy and mental resilience, alongside the willingness to invest effort and persist in the face of difficulties; dedication involves deep involvement in work accompanied by feelings of enthusiasm, inspiration, pride, and meaning; and absorption is characterized by full concentration in work activity, where time passes quickly and it is difficult to disengage from the task.

Unlike earlier conceptualizations of engagement as the opposite pole of burnout, the theoretical model underlying the UWES posits that both constructs are related but conceptually distinct, allowing for independent assessment and empirical analysis of their relationships (Schaufeli & Bakker, 2001). This distinction is central to the present study, which treats engagement as a positive well-being resource rather than merely the absence of burnout.

The original version of the instrument (UWES-17) comprises three subscales assessing the proposed dimensions: vigor (6 items), dedication (5 items), and absorption (6 items). An abbreviated nine-item version (UWES-9) was subsequently developed, with three items per dimension, aiming to improve parsimony without substantially compromising psychometric properties (Schaufeli et al., 2006). Both versions have demonstrated adequate internal consistency, with reliability coefficients typically exceeding 0.80.

Psychometric evidence indicates that while the UWES three-factor structure shows adequate fit, its constituent dimensions tend to be highly correlated. Prior studies have reported elevated inter-dimension correlations at both observed and latent levels, suggesting limited empirical differentiation among them (Schaufeli et al., 2002). Consequently, work engagement can be interpreted as either a multidimensional or unidimensional construct depending on the level of analysis and the objectives of the study.

UWES items are rated on Likert-type frequency scales, meaning that observed variables are ordinal in nature, a characteristic with direct implications for psychometric analysis, particularly regarding the choice of estimation method. Both versions of the instrument were used in the present study for comparative evaluation of their factor structures.

### 2.3. Procedure

Data was collected in the context of academic activities in three metropolitan universities, all of which are government-accredited at the advanced level and eligible for state-funded tuition-free enrollment (See Table S1: Dataset_UWES.xlsx, in Supplementary Materials). Participants were informed about the study’s objectives and provided consent prior to completing the questionnaire, ensuring voluntary participation, anonymity, and confidentiality of information. All procedures adhered to ethical standards for human research.

The sample consisted of 755 students enrolled in some of the five undergraduate programs with the highest enrollment across the three universities, and their average age was 21.6 years old (median = 20; mode = 19), suggesting a predominantly young population. However, the distribution was highly positively skewed (skewness = 4.20; kurtosis = 23.61), indicating the presence of outliers—with ages as high as 70—that considerably widen the dispersion (SD = 5.59; range = 52). These older students correspond to non-traditional entrants and continuing undergraduate education participants; sensitivity checks excluding extreme age cases yielded highly similar model patterns, supporting the robustness of the main findings. Other sociodemographic variables, such as the type of Chilean university they study at, undergraduate program, and their gender, are presented (See Table 1).

### 2.4. Data Analysis

Given that UWES items are ordinal variables, analyses were conducted explicitly accounting for this characteristic. Treating ordinal variables as continuous can affect estimation precision and the validity of conclusions in structural equation models (Li, 2016). It is also widely recognized that Likert-type data tends to deviate from multivariate normality, limiting the applicability of maximum likelihood methods under strict normality assumptions (Gao et al., 2008). Accordingly, a robust estimator appropriate for ordered categorical data was employed.

Factor models were estimated using the weighted least squares mean and variance adjusted (WLSMV) method, implemented in the lavaan package version 0.6.21 in R version 4.5.2. This estimator relies on polychoric correlation matrices and has demonstrated superior performance to maximum likelihood when analyzing ordinal variables, particularly under conditions of skewness and a limited number of response categories (Li, 2016). No missing responses were observed in the analyzed UWES items; therefore, no imputation procedure was required.

Seven factor model specifications were evaluated: unidimensional, three-factor, and second-order models for the UWES-17; unidimensional and three-factor models for the UWES-9; and bifactor models for both versions. This strategy enables direct comparison of classical CFA models with the bifactor specification, which explicitly decomposes variance into a general factor and dimension-specific factors. In the bifactor model, a general factor (g) was specified to load on all items simultaneously, alongside three orthogonal specific factors—vigor, dedication, and absorption—capturing residual variance in each dimension after controlling for the general factor. Orthogonality between the specific factors and the general factor is a defining constraint of the bifactor model that permits decoupling of both variance sources (Rodriguez et al., 2016).

Model fit was evaluated using multiple goodness-of-fit indices: chi-square (χ^2^), Comparative Fit Index (CFI), Tucker–Lewis Index (TLI), root mean square error of approximation (RMSEA), and standardized root mean square residual (SRMR). Following widely used recommendations, CFI and TLI values ≥ 0.95 and RMSEA ≤ 0.06 were considered indicative of good fit (L. T. Hu & Bentler, 1999). However, these criteria were developed under maximum likelihood estimation with continuous data, and their interpretation in models with ordinal variables should be made with caution (Xia & Yang, 2019). Accordingly, particular emphasis was placed on SRMR, which has demonstrated more robust behavior across changes in estimation method (Shi & Maydeu-Olivares, 2020).

Scale reliability was assessed using model-based composite reliability coefficients rather than the traditional alpha coefficient, as the latter assumes tau-equivalence among items, an assumption rarely met in psychological instruments and may underestimate reliability when factor loadings are heterogeneous (Cheung et al., 2023). Average variance extracted (AVE) was also computed as an indicator of convergent validity, with values above 0.50 considered acceptable evidence.

To evaluate essential unidimensionality via bifactor models, three specialized indices were calculated following Rodriguez et al. (2016): explained common variance (ECV), percent of uncontaminated correlations (PUC), and hierarchical omega (ω_h_), which estimates reliability attributable exclusively to the general factor after controlling for specific factors. Item-level ECV (I-ECV) was also computed to indicate what proportion of each item’s common variance corresponds to the general factor. The joint criterion for concluding essential unidimensionality is: ECV > 0.70, PUC < 0.80, and ω_h_ > 0.70, with these indices interpreted in combination—not in isolation—as no single index is sufficient on its own (Rodriguez et al., 2016). Indices were obtained using the BifactorIndicesCalculator package, version 01 – 2017 (Dueber, 2017) from standardized bifactor model loadings.

Finally, sex-based measurement invariance was evaluated through a sequential approach including configural, threshold-equality, and metric (loading-equality) models, appropriate for ordered-categorical indicators. Invariance was judged using changes in approximate fit indices, with ΔCFI ≤ 0.010 and ΔRMSEA ≤ 0.015 considered evidence of negligible deterioration in fit.

## 3. Results

### 3.1. Preliminary Analyses

Descriptive statistics for UWES items showed means ranging from 1.77 to 3.09 and standard deviations between 1.04 and 1.29. Skewness coefficients evidenced a general tendency toward slightly skewed distributions, particularly for items with negative skews.

Multivariate normality analysis via Mardia’s test indicated a significant violation of this assumption (skewness = 3308.93, *p* < 0.001; kurtosis = 58.54, *p* < 0.001), supporting the use of robust estimation methods for ordinal variables.

### 3.2. Comparative Model Evaluation

Seven factor models were evaluated across the UWES-17 and UWES-9: unidimensional, three-factor, and second-order for the UWES-17; unidimensional and three-factor for the UWES-9; and bifactor for both versions. This strategy enabled a comparison of classical CFA models against the bifactor specification that explicitly decomposes variance into a general factor and dimension-specific factors.

For the UWES-17, the unidimensional model showed acceptable fit on incremental indices (CFI = 0.986) but with elevated RMSEA (0.121), indicating poor global fit. The three-factor model showed a nearly identical pattern (CFI = 0.986; RMSEA = 0.120), suggesting that adding factors does not substantially improve data representation.

Critically, the second-order model failed to converge, displaying non-convergence and an inadmissible solution. This result directly indicates that the proposed hierarchical structure is incompatible with the data in the full version of the instrument.

UWES-17 models thus not only show suboptimal fit but present identification problems that limit their interpretation.

In contrast, the abbreviated version (UWES-9) showed a clearer pattern. The unidimensional model showed moderate fit (CFI = 0.993; RMSEA = 0.115), while the three-factor model evidenced consistent improvement across all indices (CFI = 0.997; RMSEA = 0.086; SRMR = 0.041), indicating that the three-dimensional structure better represents the data in this version. Within classical CFA, the three-factor model thus provides the best representation of UWES-9 scores, whereas the bifactor results reported below indicate that most reliable variance is attributable to a dominant general engagement factor, supporting the use of a total score for applied purposes. These patterns already suggest that the UWES-9 may offer a more stable structure for applied use. Fit indices for classical CFA models are presented in Table 2; bifactor models are reported in the following section alongside specialized essential unidimensionality indices.

**Table 2:** Fit indices for the UWES-17 and UWES-9 factor models ^1^.

Model	χ^2^	df	CFI	TLI	RMSEA	90% CI	SRMR	Version	Items
1F (Engagement)	1431.4	119	0.986	0.984	0.121	[0.115, 0.127]	0.076	UWES-17	17
3F (V + D + A)	1384.2	116	0.986	0.984	0.120	[0.115, 0.126]	0.076	UWES-17	17
2nd order	—	—	—	—	—	—	—	UWES-17	17
Bifactor	890.2	102	0.991	0.989	0.101	[0.095, 0.107]	0.060	UWES-17	17
1F (Engagement)	296.4	27	0.993	0.991	0.115	[0.103, 0.127]	0.059	UWES-9	9
3F (V + D + A)	157.2	24	0.997	0.995	0.086	[0.073, 0.099]	0.041	UWES-9	9
Bifactor	89.3	18	0.998	0.996	0.073	[0.058, 0.088]	0.032	UWES-9	9

^1^ Note. V = vigor; D = dedication; A = absorption. CI = confidence interval. The second-order model did not converge (inadmissible solution). Shaded rows correspond to bifactor models, which explicitly decompose variance into a general factor and specific factors; specialized essential unidimensionality indices for these models are reported in Table 3. WLSMV estimation with theta parameterization.

**Table 3:** Essential unidimensionality indices for bifactor models of the UWES-9 and UWES-17 ^1^.

Version	ECV	PUC	ω_h_	Specific ω_h_ (V/D/A)
UWES-9	0.849	0.750	0.918	0.120/0.190/≈0.000
UWES-17	0.849	0.706	0.960	0.005/0.007/0.066

^1^ Note. ECV = explained common variance of the general factor; PUC = proportion of uncontaminated correlations; ω_h_ = hierarchical omega of the general factor; Specific ω_h_ = hierarchical omega for vigor (V), dedication (D), and absorption (A) factors. Essential unidimensionality criteria (Rodriguez et al., 2016): ECV > 0.70, PUC < 0.80, ω_h_ > 0.70. Values for both versions simultaneously meet all three criteria, indicating essential unidimensionality in this sample.

Regarding the RMSEA of the UWES-9 three-factor model (0.086), its apparently elevated value should be interpreted in light of the fact that standard cutoff criteria (L. T. Hu & Bentler, 1999) were established for ML estimation with continuous variables, and that RMSEA under WLSMV tends to be systematically higher under equivalent fit conditions (Xia & Yang, 2019). The SRMR, more stable across estimation method changes (Shi et al., 2020), yielded a value of 0.041, well below the 0.05 threshold, which together with CFI = 0.997 indicates satisfactory fit.

### 3.3. Factor Discrimination in the UWES-17

Beyond fit indices, inspection of the UWES-17 three-factor model parameters revealed severe discrimination problems among factors. Specifically, the correlation between vigor and dedication exceeded unity (r = 1.014), while the correlations between vigor and absorption (r = 0.973) and between dedication and absorption (r = 0.930) also reached extremely elevated levels. A factor correlation greater than 1 indicates an improper solution, so the UWES-17 three-factor model must be considered inadmissible, and its fit indices interpreted with caution. Consequently, its global fit indices are reported for transparency but should not be interpreted as supporting the substantive validity of the three correlated factors.

This pattern constitutes evidence of factor collapse and suggests that the proposed dimensions are not empirically distinguishable in the analyzed sample. The similarity in fit indices across models therefore does not reflect theoretical equivalence, but rather a limited capacity to differentiate alternative structures.

Additionally, this factor collapse is consistent with the impossibility of estimating the second-order model, as this type of specification requires a minimum degree of differentiation among first-order factors to be identified. Furthermore, the interfactor correlations exceeding unity observed in the UWES-17 constitute direct evidence of insufficient discriminant validity among instrument dimensions: if vigor, dedication, and absorption cannot be empirically distinguished, they cannot be treated as theoretically differentiated constructs (Cheung et al., 2023).

### 3.4. Assessment of the Final Model (UWES-9, Three-Factor)

Considering fit indices, parameter stability, and solution interpretability jointly, the UWES-9 three-factor model was selected as the best representation of the data.

Standardized factor loadings were high and statistically significant in all cases, ranging from 0.722 to 0.890. Composite reliability coefficients were adequate for all three dimensions (vigor = 0.844; dedication = 0.890; absorption = 0.815), exceeding the recommended criteria. Consistently, AVE values exceeded 0.50 across all factors (vigor = 0.700; dedication = 0.780; absorption = 0.686), supporting the model’s convergent validity. These CR and AVE values should be interpreted as evidence of reliability and convergent validity within the classical three-factor CFA parameterization. However, considering the bifactor results, they should not be taken as evidence that the subscales retain substantial reliable variance independent of the general engagement factor.

Despite the expected high inter-dimension correlations, the abbreviated version thus maintains an interpretable and psychometrically adequate factor structure.

### 3.5. Measurement Invariance

Evaluation of sex-based measurement invariance yielded differentiated results across instrument versions. For the UWES-17, configural, threshold-equality, and metric models all showed similar fit indices (CFI = 0.987 across all levels), with minimal changes between models. However, these elevated indices do not constitute evidence of invariance in this case. The presence of non-positive-definite latent covariance matrices in both groups—detected across all levels of restriction—indicates that the factor solution is algebraically inadmissible. When interfactor correlations exceed unity or the latent matrix loses positive definiteness, global fit indices such as CFI can maintain apparently acceptable values because the model has sufficient free parameters to fit the data even without a valid solution. Consequently, measurement invariance cannot be reliably assessed in this version of the instrument, regardless of the reported fit index values.

The UWES-9 three-factor model, however, showed clear measurement invariance. Configural, threshold, and metric models all achieved excellent fit (CFI = 0.997 across all levels), with negligible changes across levels (ΔCFI ≤ 0.001; ΔRMSEA ≤ 0.015), supporting factorial equivalence across sex.

### 3.6. Bifactor Analysis

Bifactor models converged satisfactorily for both instrument versions and yielded the best fit indices of all models evaluated. For the UWES-9, the bifactor model obtained CFI = 0.998, TLI = 0.996, RMSEA = 0.073 [90% CI: 0.058, 0.088], and SRMR = 0.032. For the UWES-17, indices were CFI = 0.991, TLI = 0.989, RMSEA = 0.101 [90% CI: 0.095, 0.107], and SRMR = 0.060. A sensitivity analysis was conducted to evaluate whether the bifactor structure was robust to the inclusion of older participants. The UWES-9 bifactor model was re-estimated as the subsample of participants aged 30 years or younger (n = 715, 94.7% of the sample). Fit indices were virtually identical to those obtained in the full sample (CFI = 0.998, RMSEA = 0.073, SRMR = 0.033), confirming that the structural results reported are not driven by the small subset of older participants.

Specialized essential unidimensionality indices are presented in Table 3. In both instrument versions, the three criteria of Rodriguez et al. (2016) were simultaneously met: ECV > 0.70, PUC < 0.80, and ω_h_ > 0.70, constituting evidence of essential unidimensionality.

**Figure 1:**
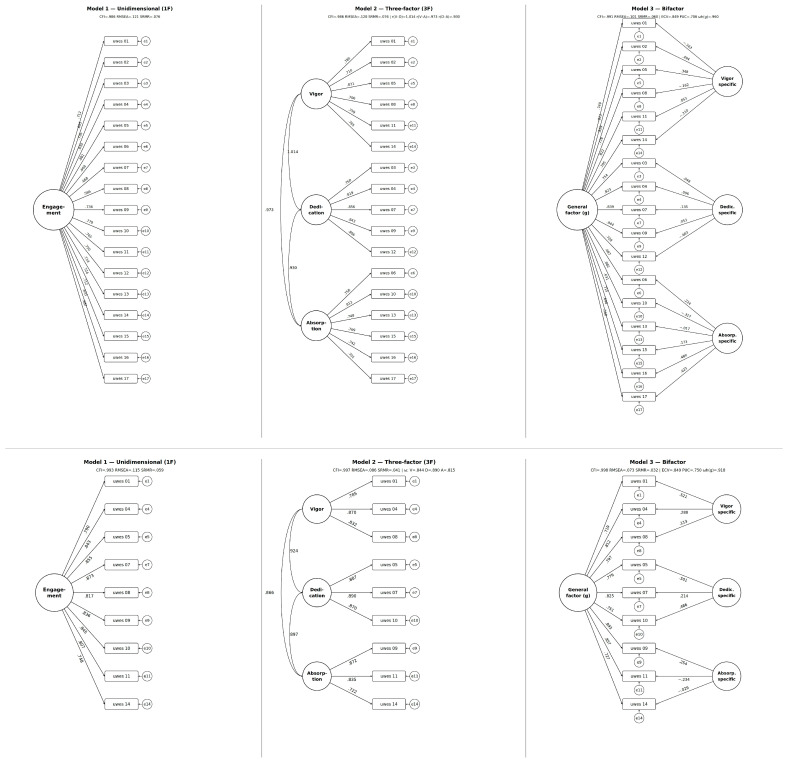
Measurement models evaluated for the Utrecht Work Engagement Scale (UWES). The upper row shows the models estimated for the UWES-17, and the lower row shows the corresponding models estimated for the UWES-9. In both versions, Model 1 represents the unidimensional solution, Model 2 the correlated three-factor solution with vigor, dedication, and absorption, and Model 3 the bifactor solution with a general engagement factor and three domain-specific factors. Standardized factor loadings are displayed on single-headed paths, curved double-headed arrows indicate latent factor correlations, and model fit indices are reported above each diagram.

General factor loadings were high and uniform across both versions, ranging from 0.568 to 0.893 in the UWES-9 and from 0.568 to 0.882 in the UWES-17. In UWES-9, specific factors for vigor and dedication retained some interpretable residual variance (ω_h_ = 0.120 and 0.190, respectively), while the absorption specific factor showed a near-zero ω_h_ (≈0.000), indicating that its items contribute no reliable variance beyond that captured by the general factor. In UWES-17, all three specific factors showed ω_h_ near zero (0.005, 0.007, and 0.066), implying that none of the dimensions retains interpretable specific variance independently of the general factor. Item uwes_12 (vigor) showed the highest specific loading in absolute value (λ = −0.683), suggesting that it constitutes a source of variance unshared with the rest of its dimension and may be contributing to the instability pattern observed in classical UWES-17 models, which underscores the need to evaluate its differential functioning in future studies (see Figure 1).

The anomalous specific loading of item 12 may be related to its distinctive content. Unlike other vigor items, which emphasize subjective energy, strength, motivation, or resilience, item 12 focuses on the ability to continue working for very long periods. In a student sample, this wording may capture endurance, workload tolerance, or prolonged effort rather than the positive energetic component of engagement. Once the general engagement factor is controlled, this content specificity may explain why the item behaves differently from the remaining vigor indicators. Therefore, the findings should be interpreted as evidence of item-level specificity or local instability, rather than as proof that the item measures an opposite construct. This interpretation is especially plausible in academic contexts, where prolonged work may reflect study demands, time pressure, or overcommitment, not only engagement.

## 4. Discussion

This study evaluated the factor structure of the Utrecht Work Engagement Scale (UWES) in its 17-item and 9-item versions in a university student sample, through systematic comparison of unidimensional, three-factor, second-order, and bifactor models. The results suggest that in this sample, the UWES is essentially unidimensional regardless of the version used. A general engagement factor accounts for 85% of common item variance in both versions (ECV = 0.849; ω_h_ > 0.90), while specific factors retain insufficient reliable variance to justify separate interpretation. These results suggest that the apparent multidimensionality of the UWES documented in literature may be, in part, a modeling artifact arising from the use of CFA specifications that do not separate general from specific variance. These structural findings have direct implications for the use of engagement measures in higher education research and practice, since they affect how institutions interpret engagement scores when monitoring student well-being.

First, results with the UWES-17 show that classical models present important limitations. Unidimensional and three-factor models had virtually identical fit indices, inter-factor correlations exceeded unity (r vigor–dedication = 1.014), and the second-order model failed to converge. The bifactor model did converge and fit better (CFI = 0.991; SRMR = 0.060), but its specialized indices revealed that the three specific factors had near-zero ω_h_ values (0.005, 0.007, and 0.066 for vigor, dedication, and absorption, respectively). This means that once the general factor variance is isolated, no reliable specific variance remains in any dimension. The factor collapse observed in classical CFA models is therefore a predictable consequence of this structure: when the general factor dominates variance so extensively, oblique three-factor solutions tend to merge. Moreover, the interfactor correlations exceeding unity in the UWES-17 constitute direct evidence of insufficient discriminant validity among instrument dimensions: if vigor, dedication, and absorption cannot be empirically distinguished, they cannot be treated as theoretically differentiated constructs (Cheung et al., 2023). In the practical context of universities, this psychometric collapse implies that designing interventions based on subscale profiles could lead to ineffective or misdirected support programs; therefore, intervention decisions should rely on indicators with demonstrated discriminant validity.

These results align with prior research questioning the multidimensional structure of the UWES, particularly in contexts where dimensions show high intercorrelations (Kulikowski, 2017). From a bifactor perspective, studies such as those of Merino-Soto et al. (2022) in Peruvian workers and Sanhokwe (2022) in Zimbabwe have consistently shown that a general factor underlies all UWES items and concentrates the majority of common variance, with the dedication dimension retaining the least specific variance independently of the general factor. The factor collapse observed in the UWES-17 in the present study is consistent with this pattern: when inter-factor correlations approach or exceed unity, first-order factors become statistically indistinguishable, and any specification (including the second-order model) becomes identification-infeasible. The evidence thus suggests that, at least in its 17-item version, the instrument may be capturing a predominantly unidimensional construct, despite its theoretical three-dimensional formulation. Moreover, situating these findings within the broader engagement literature strengthens their external validity: bifactor dominance has been observed across educational and out-of-school contexts, suggesting that a general engagement indicator may be the most robust metric for monitoring student functioning at the institutional level.

In contrast, the abbreviated version (UWES-9) showed more consistent psychometric behavior in classical models, and the bifactor model adds an important nuance to that picture. The three-factor model showed better fit than the unidimensional model, with stable parameters and adequate reliability and convergent validity. However, bifactor indices (see Table 2) show that reliable specific variance for each dimension is very limited. Vigor and dedication retain some residual specific variance (ω_h_ = 0.120 and 0.190, respectively), but absorption has ω_h_ ≈ 0.000, indicating that its three items are practically redundant with the general factor without contributing reliable specific variance. This pattern constitutes evidence of insufficient discriminant validity for absorption: this dimension cannot be empirically distinguished from the general engagement factor in this sample. This nuance supports the pragmatic adoption of the UWES-9 total score in higher education settings where brevity and reliability are priorities for large-scale well-being assessments, offering a favorable balance between measurement efficiency and psychometric robustness.

These results are consistent with those of Merino-Soto et al. (2022) in Peruvian workers and Sanhokwe (2022) in Zimbabwe, who found the same pattern of general factor dominance. This pattern aligns with broader engagement research showing that bifactor structures consistently reveal a strong general engagement factor across school, afterschool, and university contexts (Shernof et al., 2017; Sjogren et al., 2022). This study extends that evidence to a Chilean university student sample, suggesting that the essential unidimensionality of the UWES may extend beyond the present Chilean university sample. However, further cross-cultural, occupational, and longitudinal replications are needed before treating this pattern as context independent. Additionally, the finding that dedication retains slightly more specific variance than vigor in the UWES-9, while in the UWES-17 this dimension is almost entirely absorbed by the general factor, suggests that the relative stability of the three-factor structure in the abbreviated version is partly due to the item selection process for the short form, which tends to retain items with lower inter-dimension shared variance.

A key implication concerns how UWES scores should be computed and interpreted. Given that the general factor dominates variance in both instrument versions and that specific factors do not retain sufficient reliable variance—especially absorption, whose ω_h_ was near zero in both versions—the use of a total engagement score is empirically justified. Computing separate subscores for vigor, dedication, and absorption not only lacks empirical support but may introduce interpretive errors by attributing between-dimension variability that is general factor variance. This recommendation is consistent with that of other authors following bifactor analysis of the UWES (Merino-Soto et al., 2022; Sanhokwe, 2022), and with the practice that Schaufeli et al. (2006) themselves acknowledged as valid when computing a single total score. From a policy perspective, recommending a single overall score may simplify future monitoring systems by reducing the interpretive ambiguity associated with unstable subscale profiles. However, the present study provides structural validity evidence only. Therefore, the use of UWES-9 scores for screening, operational thresholds, follow-up protocols, or referral pathways should be considered a potential application that requires additional criterion-related, predictive, and sensitivity-to-change evidence.

These results also carry methodological implications for CFA in multidimensional instruments with high inter-factor covariation. Classical CFA model comparisons (1F, 3F, 2nd order) may be insufficient when dimensions are highly correlated, as fit indices tend to be similar across competing models and factor collapse may go undetected. This limitation is consistent with broader engagement research showing that CFA tends to inflate factor correlations and obscure multidimensionality (Lee, 2021). More broadly, these findings suggest that the apparent multidimensionality of the UWES may be in part a methodological artifact: classical CFA models assume that factors are the only source of shared item variance, and when that variance is predominantly common to all items, the three-factor model produces fit very similar to the unidimensional model precisely because both are capturing essentially the same thing. Incorporating the bifactor model allows going beyond global fit and directly quantifying how much variance is general versus specific to each dimension, providing a direct answer to the question of the construct’s dimensionality. For applied researchers in higher education, we recommend routinely reporting bifactor indices (e.g., ECV, ω_h_, PUC) and using robust ordinal estimators (e.g., WLSMV) alongside ML estimators to ensure informed scoring decisions aligned with the study’s well-being objectives.

Beyond the methodological contribution, this finding has a direct theoretical implication: the distinction between vigor, dedication, and absorption as conceptual differentiated components of engagement does not hold empirically when a modeling framework is applied that decouples general from specific variance. This does not imply that the three-dimensional theory of engagement is incorrect. Rather, it indicates that the current UWES items do not capture these distinctions with sufficient empirical precision, limiting the available psychometric evidence to support their operationalization as distinct dimensions. Theoretically, this suggests that research on engagement in higher education should more clearly distinguish between when engagement is conceptualized as an indicator of well-being (useful for monitoring and policy-making), and when it is viewed as a multidimensional construct for theoretical research or targeted interventions.

Measurement invariance analyses showed consistent structural equivalence by sex for the UWES-9; UWES-17 invariance assessment remains limited by the identification problems discussed above. This suggests that the abbreviated version not only presents better psychometric properties but is also more adequate for between-group comparisons. This invariance facilitates demographic comparisons in institutional wellness programs and allows for the evaluation of the effectiveness of interventions by sex without measurement bias.

Collectively, these findings contribute directly to the debate on UWES dimensionality. Bifactor evidence shows that engagement, in both instrument versions, is an essentially unidimensional construct in this sample: a single general factor captures the large majority of common variance, specific dimensions contribute very little additional reliable variance, and absorption retains practically null specific variance across both versions. Thus, the UWES-9 three-factor model should be understood as the best-fitting and most stable solution within the family of classical CFA models. However, the bifactor indices indicate that the reliable variance captured by these factors is largely attributable to a dominant general engagement factor, which supports prioritizing the total score over separate subscale interpretation. These results are consistent with those of Merino-Soto et al. (2022) and Sanhokwe (2022) in other cultures and populations, suggesting that the essential unidimensionality of the UWES is a robust finding independent of cultural context or sample type, although broader generalization across cultural and occupational contexts will require additional replications. Consequently, interventions aimed at increasing overall engagement may yield broader benefits for academic outcomes and student well-being than interventions narrowly targeted at putative subdimensions that the instrument cannot reliably distinguish.

### 4.1. Practical Implications

These findings have direct implications for three types of UWES users: academic researchers, organizational and clinical practitioners, and instrument developers. In all three cases, evidence of essential unidimensionality justifies modifying standard instrument use practices and provides a parsimonious explanation for the ambiguity that has characterized the literature on its dimensionality. Additionally, we recommend that studies intended to inform university policy include predictive validity analyses linking the UWES total score to mental health indicators, retention, and academic performance to demonstrate practical utility for institutional decision-making.

#### 4.1.1. Recommendations for Researchers

The first recommendation is to use the UWES-9 total score as the engagement measure rather than computing separate subscales. Given that specific factors do not retain sufficient reliable variance (especially absorption), computing three-dimensional scores add no valid information and may introduce interpretive error. This is consistent with the practice that the instrument’s own authors acknowledged as valid (Schaufeli et al., 2006), but that has not been the norm in empirical literature. These findings support the use of the full UWES-9 as an institutional monitoring tool and underscore the need to link psychometric measurements with strategies for promoting student mental health. At the institutional level, we suggest a simple operational protocol: (1) administer the UWES-9 total score on a regular basis (e.g., biannual surveys); (2) define operational thresholds for follow-up; and (3) link threshold breaches to clear referral pathways for psychological support and well-being programs.

The second recommendation concerns the interpretation of prior literature. The historical ambiguity in UWES dimensionality findings, where approximately half of studies support the three-factor model and the other half support the unidimensional model (Kulikowski, 2017), does not reflect genuine variability across contexts or samples but is a predictable consequence of the instrument’s bifactor structure: when the general factor extensively dominates variance, classical CFA models of one and three factors tend to produce similar and interchangeable fit indices. Recognizing this explanation allows for more precise reading of prior literature and more methodologically rigorous study comparisons.

The third recommendation is methodological: studies modeling UWES subscales as separate predictors or criteria in regression analyses or structural equation models should be interpreted with caution. Given that the three dimensions share most of their variance through the general factor, their simultaneous inclusion as independent variables introduces substantial multicollinearity, which can distort the estimated coefficients and impede an accurate assessment of each dimension’s specific effects.

#### 4.1.2. Recommendations for Organizational and Clinical Practitioners

In applied contexts, the UWES-9 total score is sufficient and more stable than subscales for assessing an individual or group’s general engagement level. The instrument in its current form does not have the psychometric specificity to support assertions such as “this student has high vigor but low absorption”: since absorption contributes no reliable specific variance beyond the general factor, that difference between subscales would reflect measurement noise rather than a genuine individual characteristic. Engagement improvement interventions should therefore be designed based on the general construct level, and not on dimensional profiles that the instrument cannot reliably distinguish. At the institutional level, the UWES-9 total score could be incorporated into regular surveys as a general indicator of academic engagement. Nevertheless, before defining operational thresholds, follow-up criteria, or referral pathways, future studies should test its criterion-related validity, predictive utility, and sensitivity to change in relation to mental health, retention, academic performance, and well-being outcomes. This recommendation is consistent with prior bifactor analyses of the UWES and with broader engagement research showing that the general engagement factor is the strongest predictor of academic outcomes across educational contexts (Shernof et al., 2017; Sjogren et al., 2022).

The evidence of metric sex-based invariance obtained in this study further indicates that the UWES-9 total score is comparable across male and female participants, making it suitable for institutional diagnostics requiring demographic group comparisons. This property, combined with the robustness of the general factor, supports the instrument’s use in large-scale assessments in educational and organizational settings.

#### 4.1.3. Recommendations for Future Instrument Development

If the theoretical and applied objective requires measuring vigor, dedication, and absorption as genuinely distinct constructs, the UWES in its current form is not the appropriate instrument for that purpose. Achieving real dimensional differentiation would require developing new items with greater specific variance, particularly for absorption, whose items in both versions are practically interchangeable with the general factor. This finding suggests that current absorption items do not capture the cognitive component of task concentration in a sufficiently specific manner, possibly because their wording conceptually overlaps with the motivational component that defines the general factor. If the institutional goal is to assess specific dimensions for targeted interventions, we recommend a collaborative item development process involving university mental health professionals, pedagogical experts, and students, followed by pilot testing that includes clinical and academic performance criteria.

In its current state, the UWES-9 total score appears to be the most defensible scoring option for the present type of application: it maximizes general factor reliability (ω_h_ = 0.918), minimizes error from unstable subscale estimation, and is the only score with sufficient structural support for between-group comparisons in this sample. Its systematic adoption in research and organizational practice would contribute to reducing the methodological fragmentation that has impeded the accumulation of comparative evidence on engagement across diverse contexts. Widespread adoption of the UWES-9 total score would also enable the construction of longitudinal series that are valuable for evaluating the effectiveness of well-being policies and detecting population-level trends in student mental health.

### 4.2. Limitations and Future Research Directions

Several limitations of this study should be acknowledged. First, the sample is limited to university students from Metropolitan Region of Chile, constraining generalizability to other populations and occupational settings; replication in worker samples and other Latin American contexts is needed. Second, the cross-sectional design precludes an assessment of temporal stability; given that engagement may fluctuate across academic and work conditions, longitudinal replication of the essential unidimensionality finding would be informative. Third, item uwes_12 showed an unusually high and negative specific loading (λ = −0.683) on the dedication factor in the UWES-17 bifactor model, suggesting that this item may be functioning discrepantly relative to the rest of its dimension in this sample. The large negative specific loading observed for this item suggests that it may capture item-specific variance or local instability after the general factor is controlled. This pattern may partly contribute to the instability of the UWES-17 models, but it should not be overinterpreted without item-level follow-up analyses, such as DIF or IRT-based evaluation. Future research should examine the differential item functioning of this item and consider whether its inclusion in the scale is psychometrically advisable in student populations.

Future work should replicate the bifactor analysis in Chilean worker samples to enable direct comparison. IRT-based differential item functioning analysis of uwes_12 would clarify whether its atypical behavior is sample-specific or reflects a broader content issue. Finally, examining whether the UWES general-factor total score predicts relevant outcomes (academic performance, well-being, burnout) as well as or better than subscales would provide criterion validity evidence complementing the structural findings reported here. Importantly, the present study provides structural validity evidence but does not directly test criterion-related validity, predictive validity, or sensitivity to change. Therefore, applied uses of the UWES-9 total score for institutional monitoring, screening, or program evaluation should be considered provisional until supported by external validity evidence. Furthermore, future research should assess the sensitivity of the UWES total score to changes induced by well-being interventions (i.e., sensitivity to change) and explore its utility as a screening tool to complement clinical measures of anxiety and depression in university settings.

## 5. Conclusions

These findings indicate that in this Chilean university sample, work engagement as measured by the UWES behaves largely as a single, overarching construct rather than three clearly separable dimensions. Bifactor analyses show that a dominant general engagement factor accounts for the vast majority of shared item variance (ECV ≈ 0.85; ω_h_ > 0.90), while the specific subscales (vigor, dedication, absorption) contribute little reliable unique variance. In practical terms, this means that reporting a single UWES total score captures the construct reliably and avoids misleading interpretations based on unstable subscores.

We therefore recommend using the UWES-9 total score for routine assessment of general academic engagement in higher education, because it offers a concise and psychometrically defensible indicator in this Chilean university sample. This recommendation is grounded primarily in structural validity evidence and is pragmatic rather than theoretical: it does not deny the conceptual value of vigor, dedication, and absorption, but it recognizes that with the current items, these dimensions are not empirically distinct enough to be treated as independent scales in applied settings.

Finally, the study highlights implications for practice and future research. Universities that use engagement measures for population monitoring or program evaluation may benefit from prioritizing the total score over unstable subscale profiles. However, the use of UWES-9 scores for screening, operational thresholds, referral decisions, or policy evaluation should be considered a potential application pending criterion-related, predictive, and sensitivity-to-change evidence. Future research should evaluate the scale’s predictive validity for academic and mental-health outcomes, its responsiveness to change, and its utility for institutional decision-making. Further refinement of the item pool is also needed to determine whether more reliable and actionable subscales can be developed to support targeted interventions and student well-being monitoring.

## Figures and Tables

**Table 1:** Sociodemographic characterization of the student sample.

Variable	Category	Sample	% Sample
Students by type of Chilean Universities	Public University	221	29.27%
	Secular Private University	279	36.95%
	Catholic Private University	255	33.78%
Student by Undergraduate Program	Accounting/Auditing	73	9.67%
	Business Administration	157	20.79%
	Industrial Engineering	154	20.40%
	Law	193	25.56%
	Psychology	178	23.58%
Students by Gender	Female	380	50.33%
	Male	375	49.67%

## Data Availability

The original contributions presented in this study are included in the Supplementary Material. Further inquiries can be directed at the corresponding authors.

## Supplementary Materials

The following supporting information can be downloaded at: https://www.mdpi.com/article/10.3390/bs16071045/s1, Table S1: Dataset_UWES.xlsx.

## Abbreviations

The following abbreviations are used in this manuscript:

AVE	Average Variance Extracted
CFA	Confirmatory Factor Analysis
CFI	Comparative Fit Index
ECV	Explained Common Variance
IRT	Item Response Theory
PUC	Percent of Uncontaminated Correlations
RMSEA	Root Mean Square Error of Approximation
SD	Standard Deviation
SRMR	Standardized Root Mean Square Residual
TLI	Tucker–Lewis Index
UWES	Utrecht Work Engagement Scale
WLSMV	Weighted Least Squares Mean and Variance Adjusted
